# Efficacy of swm appliance in the expression of first-, second- and third-order information in Class I and Class II

**DOI:** 10.4317/jced.55399

**Published:** 2019-01-01

**Authors:** Luca Lombardo, Michele Calabrò, Virginia Squarci, Anna Colonna, Giuseppe Siciliani

**Affiliations:** 1 Department of Orthodontics, University of Ferrara, Ferrara, Italy. Via Montebello 31, 44100 Ferrara, Italy; 2 Private Practise Via Carlo Pezzani, 37, 27058 Voghera PV; 3 Chairman. Department of Orthodontics, University of Ferrara, Ferrara, Italy. Via Montebello 31, 44100 Ferrara, Italy

## Abstract

**Background:**

To assess the efficacy of a multibracket appliance—Straight-wire Mirabella (SWM) prescription—in terms of achieving the ideal first-, second- and third-order values proposed by Andrews.

**Material and Methods:**

A total sample of 46 Caucasian subjects was divided into two groups: 23 with class I malocclusion (Group 1), and 23 with class II malocclusion (Group 2). The treatment protocol involved fixed multibracket appliances—SWM prescription—for both groups, with the addition of class II elastics for Group 2. Values for ΔU1-PP, ΔIMPA, in-out, tip and torque were measured on digital scans, and the results obtained were compared with the ideal values proposed by Andrews.

**Results:**

Statistically significant differences were revealed between the entire sample and Andrews’ values for: in-out on upper lateral incisors and upper canines; tip on the upper first premolars, upper second premolars, upper first molars and upper canines; and torque on the lower central incisors, lower lateral incisors, lower canines and lower first premolars. However, comparison of Groups 1 and 2 revealed statistically significant differences only at the lower lateral incisors. The use of class II elastics influenced ΔIMPA values, but not ΔU1-PP.

**Conclusions:**

The efficacy of the multibracket appliance—SWM prescription—in expressing first- second- and, to a lesser extent, third-order information was demonstrated in both class I and class II malocclusions. Class II elastics only influenced the third-order expression on the lower lateral incisors and the ΔIMPA.

** Key words:**Straight wire fixed appliances, prescription efficacy, Class I malocclusions, Class II malocclusions.

## Introduction

Dr. Andrews study (1) regarding treatment outcomes in terms of the ideal first-, second- and third-order values for optimal occlusion had a significant impact in Orthodontics. Once the first prescription had been introduced, many modifications were proposed with the aim of minimising the need for bending during the finishing stages of treatment. Despite all this attention, however, the actual post-treatment in-out, tip and torque often differ from those expected (2). Indeed, there are several mechanical factors that limit the complete expression of first-, second- and third-order information, and therefore make compensatory measures essential, especially in terms of torque (2), when using pre-programmed appliances. Limiting factors are linked to the structural characteristics of both archwire and bracket; in particular, the mesiodistal distance between brackets has a particularly strong influence on the tip (3) while torque is affected by the dimensions of the archwire and slot, which, as demonstrated on several occasions, often differ from those claimed by the manufacturers (4,5) and therefore make the actual play different from the ideal (6). Another major influence on the expression of prescription is the use of auxiliaries such as class II elastics—one of the most commonly used in the correction of class II (7). In this regard, a recent review of the literature (8) has suggested that many of the collateral effects of these auxiliaries may be different to those reported (9) In order to clarify the issue, the aim of this study was to assess the efficacy of the SWM prescription in terms of achieving the ideal first-, second- and third-order values proposed by Andrews, 1 and to evaluate variations in the expression of ΔU1-PP and ΔIMPA values, in particular any differences associated with the use of class II elastics.

## Material and Methods

A total sample of 46 Caucasian subjects that met the following inclusion criteria was selected.

• Permanent dentition

• Class I or II malocclusion

• Availability of pre- and post-treatment laterolateral teleradiographs, panoramic radiographs and photographs 

The exclusion criteria were:

• Dental anomalies

• Prosthetic restorations or implants

• Extractions

• Little’s index greater than 5

• Stripping

The sample (mean age 12.37 ± 2.07) was then divided into two treatment groups.

• Subjects presenting class I (Group 1: 10 males and 13 females of mean age 12.73 ± 2.27)

• Subjects presenting class II (Group 2: 8 males and 15 females of mean age 12.02 ± 1.84)

Patients were treated by two different operators—both Orthodontics specialists certified by the EBO (European Board of Orthodontists)—using fixed multibracket appliances, SWM prescription. The archwire sequence for each patient was as follows:

• .016 NiTi thermoactive

• .019x.025 NiTi thermoactive

• .019x .025 stainless steel

The following parameters were measured for each patient:

• In-out (first order)

• Tip (second order)

• Torque (third order)

• ΔIMPA: variation in inclination of lower incisors with respect to the mandibular plane 

• ΔU1-PP: variation in inclination of upper incisors with respect to the palatal plane

The parameters of interest were measured by the following procedure:

• Models were scanned using a 3Shape R500 scanner (3Shape, Copenaghen) 

• In-out, tip and torque of each tooth were measured using VAM software (Vectra, Canfield Scientific, Fairfield, NJ, USA) according to the method proposed and validated by Luis Huanca10, expanded to 100 points per model.

• Cephalometric analysis was performed via Delta-Dent software (Outside Format, Spino D’Adda, CR, Italy) in order to evaluate ΔIMPA and ΔU1-PP

The post-treatment values for the above parameters were compared between groups and with those proposed by Andrews. Statistical analysis was performed using SAS 9.3 statistical software as follows:

• Repeatability analysis: performed after 6 weeks by replicating the measurements on 11 models randomly selected from the total of 46. The two datasets were used to evaluate the method repeatability by calculating their mean absolute error and standard deviation, Dahlberg’s index, Bland-Altman plots and generalised estimating equation (GEE) linear regression.

• Continuous variables were compared between groups using Student’s t (with or without Satterthwaite’s adjustment, as appropriate) or Mann-Whitney tests, after assessing their normal distribution, by means of Shapiro-Wilks, and variance equality via the F test.

• *P*-values for multiple comparisons were corrected using the False Discovery Rate.

• The effects of pre-treatment IMPA, class II elastics, Δ LITTLE and Δ SPEE on Δ IMPA were evaluated using a multiple linear regression model.

• The statistical significance threshold was set at *p*<0.01.

## Results

-Repeatability analysis

The mean absolute error values for the measurements of first-, second- and third-order expression are reported in  Table 1.

**Table 1:**
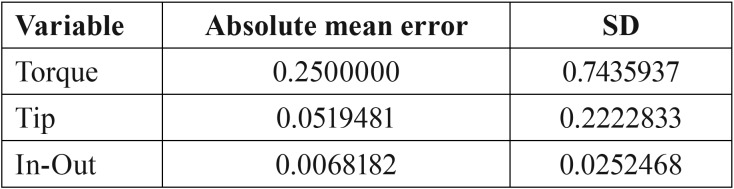
Absolute mean error and standard deviation for first-, second-, and third-order measurements.

-First order (in-out)

The results for first-order expression are shown in  Table 2. The following statistically significant differences (*P* FDR <.01) were found:

**Table 2:**
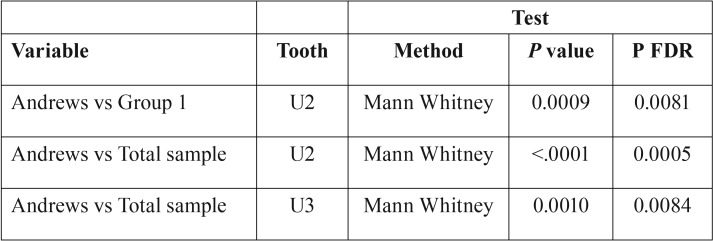
Comparison of in-out values (U = Upper, L= Lower, *** = *P* value <.001, ** = *P* value <.01, * = *P* value <.05).

• Entire sample vs. Andrews:

o Upper lateral incisors: 1.6 mm ± 0.2 mm vs. 1.8 mm ± 0.2 mm; mean difference 0.2 mm

o Upper canines: 2.5 mm ± 0.2 mm vs. 2.7 mm ± 0.3 mm; mean difference 0.2 mm

• Group 1 vs. Andrews:

o Upper lateral incisors: 1.6mm ± 0.3mm vs. 1.8mm ± 0.2mm; mean difference 0.2mm

• No statistically significant differences were found in comparisons of Group 2 vs. Andrews, or Group 1 vs. Group 2.

-Second order (tip)

The results for second-order expression are shown in  Table 3. The following statistically significant differences (P FDR <.01) were found:

**Table 3:**
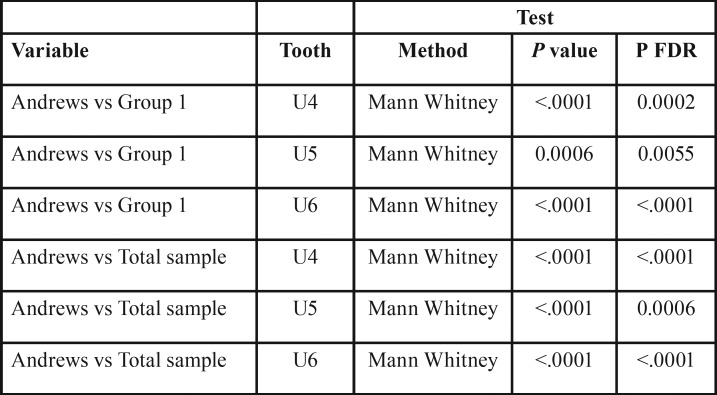
Comparison of tip values (U = Upper, L= Lower, *** = *P* value <.001, ** = *P* value <.01, * = *P* value <.05)

• Entire sample vs. Andrews:

o Upper first premolars: 1.8° ± 1.2° vs. 2.9° ±1.6°; mean difference 1.1°

o Upper second premolars: 2° ± 1.7° vs. 2.9° ±1.2; mean difference 0.9°

o Upper first molars: 3.7° ± 2.3° vs. 5.7° ±1.6°; mean difference 2°

• Group 1 vs. Andrews:

o Upper first premolars: 2.3° ± 1.3° vs. 2.9° ± 1.6°; mean difference 0,6°

o Upper second premolars: 2.5° ± 1.4° vs. 2.9° ± 1.2°; mean difference 0.4°

o Upper first molars: 4.5° ± 1.8° vs. 5,7° ± 1.6°; mean difference 1.2°

• No statistically significant differences were found in comparisons of Group 2 vs. Andrews, or Group 1 vs. Group 2.

-Third order (torque)

The results for third-order expression are shown in  Table 4. The following statistically significant differences (P FDR <.01) were found:

**Table 4:**
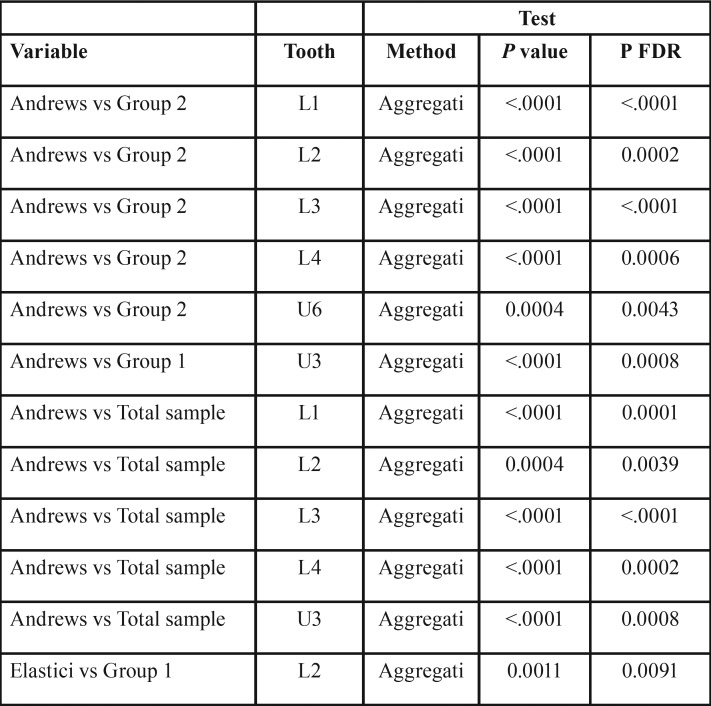
Comparison of torque values (U = Upper, L= Lower, *** = *P* value <.001, ** = *P* value <.01, * = *P* value <.05).

• Entire sample vs. Andrews:

o Upper canines: -7.3° ±3.7° vs. -4.5° ±4.5°; mean difference 2.8°

o Lower central incisors: -1.7° ±5.7° vs. 3° ±5.4°; mean difference 4.7°

o Lower lateral incisors: -3.2° ±5.3° vs. 0.1° ±4.8°; mean difference 3.3°

o Lower canines: -12.7° ±4.3° vs. -8.6° ±4°; mean difference 4.1°

o Lower first premolars: -18.9° ±4.5° vs. -15.5° ±4.2°; mean difference 3.4°

• Group 1 vs. Andrews:

o Upper canines: -3.6° ± 4,9° vs. -7,3° ± 3.7°; mean difference 3.7°

• Group 2 vs. Andrews:

o Upper first/second molars: -14.5° ± 39° vs. -11.5° ± 3.5°; mean difference 3°

o Lower central incisors: 5.2° ± 54° vs. -1,7° ± 5,7°; mean difference 6.9°

o Lower lateral incisors: 2.3° ± 4.5° vs. -3.2° ± 5.3°; mean difference 5.5°

o Lower canines: -7.4° ± 3.9° vs. -12.7° ± 4.3°; mean difference 5.3°

o Lower first premolars: -14.6° ± 4.4° vs. -18.9° ± 4.5°; mean difference 4.3°

• Group 1 vs. Group 2:

o Lower lateral incisors: -2.1°± 4.1° vs. – 2.3°±4.5°; mean difference 4.4°

-ΔIMPA 

The mean differences found between Groups 1 and 2 were:

• Pre-treatment IMPA: 5.5°

• Post-treatment IMPA: 7.7°

• ΔIMPA 2.3°

Multivariate analysis of ΔIMPA and the use of class II elastics, variation in the curve of Spee, Little index, and pre-treatment IMPA revealed statistically significance influences for class II elastics and pre-treatment IMPA, but not variation in the curve of Spee or Little index ( Table 5.

**Table 5:**
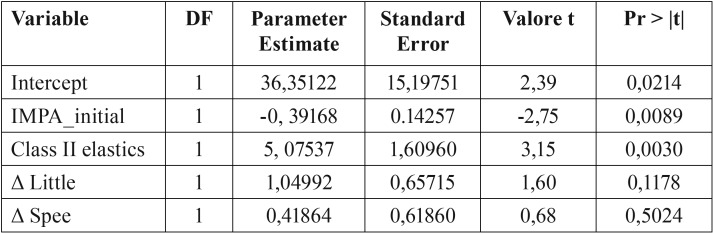
Multivariate analysis between Δ IMPA and: initial IMPA, class II elastics, Δ LITTLE and Δ SPEE.

-ΔU1-PP 

The mean differences found between Groups 1 and 2 were:

• Pre-treatment U1-PP: 3.6°

• Post-treatment U1-PP: 0.4°

• ΔU1-PP: -3°.

The difference between Groups 1 and 2 was not found to be statistically significant ( Table 6).

**Table 6:**
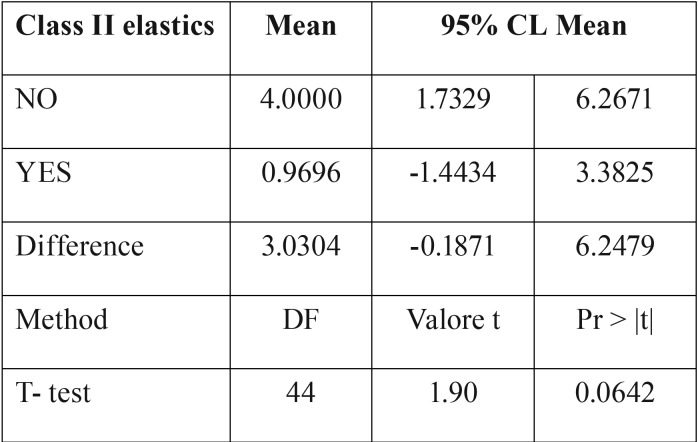
T-test assessment of ΔU1-PP values in Groups 1 and 2.

## Discussion

In order to compare our first-, second- and third-order values with those reported by Andrews,1 we used VAM software (10) (Vectra, Canfield Scientific, Fairfield, NJ, USA) to measure in-out, tip and torque. We decided to use a digital method of measurement due to the various advantages that this presents, namely greater precision, rapidity of execution, and the possibility to superimpose data derived from manual measurement systems (11-13). Indeed, our digital measurements were in good agreement, displaying a mean error similar to (but slightly lower than) that described by Huanca (10) 

A key factor in obtaining precise measurements was the definition of the occlusal plane, which remained stable and unvarying for each measurement of tip and torque (10). As regards in-out ( Table 2), the differences found between our entire sample and Andrews’ (1) may be ascribed to the different measurement methods employed. Indeed, Andrews used a sharpened Boley gauge to measure the vestibular prominence of the crowns after removing their occlusal halves, tracing the embrasure line (a line uniting the most vestibular points of the contact areas on the vestibular surface) (1), while our measurements were made considering the mesial and distal points (incisiors and canines) and the points on the mesial and distal marginal crest (premolars and molars), thereby obtaining a segment from which the distance from the FA point could be calculated (10). In other words, the occlusal halves were not removed, and rather than a single embrasure line, each tooth was assessed separately. Although this difference in methodology did result in small discrepancies, these differences were not clinically significant.

Likewise, the differences in second-order ( Table 3) between our sample and Andrews’ values,1 specifically at the upper first premolars, upper second premolars and upper first molars, were not clinically significant, even though the statistical significance threshold was reached. Greater differences between ours and Andrews’ samples were, however, found for the third-order values, which were statistically significant for several teeth ( Table 4). That being said, the lack of discrepancy observed at the upper incisors confirms the efficacy of the appliance used, which was able to provide sufficient torque to the upper anterior sector even with almost 10° of play between the .022x.028 slot and the .019x.025 archwire (14); this was made possible thanks to a prescription of 17° for the upper central incisors and 8° for the upper lateral incisors.

It was interesting to note that the upper canines, particularly in Group 1, presented a less negative post-treatment torque than the values reported by Andrews (-7.3° vs. -3.6°). This can be ascribed to the prescription, which was designed to produce 0° at the upper canines, making the -7° value optional. Of note, the only statistically significant difference between our Group 1 and Andrews’ sample was at these teeth.

As regards the differences between our Group 2 and Andrews’ sample (1)—found at the upper molars and incisors, and lower canines, incisors and first premolars—it appears evident from the study design that these were ascribable to the use of class II elastics. Our data suggest that class II elastics have a greater effect on third-order expression than on tip and in-out. Indeed, when comparing our Group 1 and 2, the only statistically significant differences we found were in terms of torque and ΔIMPA, suggesting that these auxiliaries have only a partial influence on the expression of the prescription inserted into the brackets. This concept is further confirmed by the fact that the differences we found between our Group 2 and Andrews’ sample1 were more substantial than those between the latter and our entire sample. Furthermore, it has been amply documented in the literature that elastics lead to proclination of the lower incisor group (7,14).

Our data suggest that this proclination is greater at the lower central, rather than lateral, incisors, and that this difference in torque between central and lateral incisors was maintained in both groups. Indeed, The lower lateral incisors present 2° more coronolingual torque than the central incisors in the same arch, a feature previously noted by Andrews1. This is why the SWM prescription is designed to provide a more negative torque on the lower lateral incisors (-10°) with respect to their central counterparts (-6°).

The more positive mean final torque on the lower incisors presented by our Group 1 with respect to Andrews sample1 may be due to the fact that the latter group comprised subjects in ideal occlusion, whereas our Group 1 patients had been treated to resolve class I malocclusion, in which crowding correction (even if minimal due to the exclusion of patients with a Little’s index above 5) and flattening of the curve of Spee (15) play a fundamental role in proclination of the lower incisor group.

The variation between pre-and post-treatment torques was not considered in either Group in this study, as the change in inclination of the occlusal plane brought about by class II elastics would have influenced the comparison. That being said, the literature does not indicate a consensus on the actual effects of these auxiliaries on the upper incisors. Nelson, for example, has published several articles on the dental and skeletal effects of class II elastics, with contrasting results (14,16,17). Our data indicate a lesser torque on the central incisors in Group 1 with respect to Group 2, while the torque on the lateral incisors was comparable between the two Groups. Nevertheless, the difference we found was not statistically significant, indicating that the class II elastics were not associated with post-treatment retroclination of the upper incisor group.

In summary, the prescription considered was found to be extremely efficacious in terms of in-out and tip expression in both subgroups and the sample as a whole. The greatest differences between our treatment groups and Andrews ideal sample1 were, in fact, measured for torque; however, in addition to the abovementioned considerations, this was probably due to the influence of several factors on torque expression, including the archwire edge bevel, the position of the brackets with respect to the tooth morphology, the ligature system employed, and the initial inclination of the teeth (18,19). As demonstrated, these factors often make it necessary to resort to archwire bending in order to achieve ideal torque values.

## Conclusions

The efficacy of the multibracket appliance—SWM prescription—in expressing first- second- and third-order information was demonstrated in both class I and class II malocclusions. The greatest differences with respect to Andrews’ ideal values were slight in both groups, reaching statistical significance only at the lower lateral incisors. Class II elastics only influenced the third-order expression on the lower lateral incisors and the ΔIMPA.

## References

[1] Andrews F (1972). The six keys to normal occlusion. Am J Orthod.

[2] Siatkowski RE (1999). Loss of anterior torque control due to variations in bracket slot and archwire dimensions. J Clin Orthod.

[3] Kusy RP, Whitley JQ (1999). Assessment of second-order clearances between orthodontic archwires and bracket slots via the critical contact angle for binding. Angle Orthod.

[4] Cash AC, Good SA, Curtis RV, McDonald F (2004). An evaluation of slot size in orthodontic brackets—are standards as expected?. Angle Orthod.

[5] Arreghini A, Lombardo L, Mollica F, Siciliani G (2014). Torque expression capacity of 0.018 and 0.022 bracket slots by changing archwire material and cross section. Progress in Orthodontics.

[6] Lombardo L, Arreghini A, Bratti E, Mollica F, Spedicato G, Merlin M (2015). Comparative analysis of real and ideal wire - slot play in square and rectangular archwires. Angle Orthod.

[7] Jones G, Buschang PH, Kim KB, Oliver DR (2008). Class II non-extraction patients treated with the Forsus fatigue resistant device versus intermaxillary elastics. Angle Orthod.

[8] Janson G, Sathler R, Fernandes TMF, Cabral Castello Branco N, De Freitas MR (2013). Correction of Class II malocclusion with Class II elastics: A systematic review. Am J Orthod Dentofacial Orthop.

[9] Ellen EK, Schneider BJ, Sellke T (1998). A comparative study of anchorage in bioprogressive versus standard edgewise treatment in Class II correction with intermaxillary elastic force. Am J Orthod Dentofacial Orthop.

[10] Huanca Ghislanzoni LT, Lineberger M, Cevidanes LHS, Mapelli A, Sforza C, McNamara JA Jr (2013). Evaluation of tip and torque on virtual study models: a validation study. Progress in Orthodontics.

[11] Naidu D, Scott J, Ong D, Ho CTC (2009). Validity, reliability and reproducibility of three methods used to measure tooth widths for Bolton analyses. Aust Orthod J.

[12] Dalstra M, Melsen B (2009). From alginate impressions to digital virtual models: accuracy and reproducibility. J Orthod.

[13] Fleming PS, Marinho V, Johal A (2011). Orthodontic measurements on digital study models compared with plaster models: a systematic review. Orthod Craniofac Res.

[14] Nelson B, Hansen K, Hagg U (1999). Overjet reduction and molar correction in fixed appliance treatment of Class II, Division 1, malocclusions: sagittal and vertical components. Am J Orthod Dentofacial Orthop.

[15] Pandis N, Polychronopoulou A, Sifakakis I, Makou M, Eliades T (2010). Effects of levelling of the curve of Spee on the proclination of mandibular incisors and expansion of dental arches: a prospective clinical trial. Aust Orthod J.

[16] Nelson B, Hagg U, Hansen K, Bendeus M (2007). A long-term follow-up study of Class II malocclusion correction after treatment with Class II elastics or fixed functional appliances. Am J Orthod Dentofacial Orthop.

[17] Nelson B, Hansen K, Hagg U (2000). Class II correction in patients treated with Class II elastics and with fixed functional appliances: a comparative study. Am J Orthod Dentofacial Orthop.

[18] Morina E, Eliades T, Pandis N, Jäger A, Bourauel C (2008). Torque expression of self-ligating brackets compared with conventional metallic, ceramic, and plastic brackets. European J Orthod.

[19] Archambault A, Lacoursiere R, Badawi H, Major PW, Carey J, Flores-Mir C (2010). Torque expression in stainless steel orthodontic brackets. A systematic review. Angle Orthod.

